# Supplementary material for: Early impact of agropastoral activities and climate on the littoral landscape of Corsica since mid-Holocene
Source: PLoS One. 2019 Dec 19;14(12):e0226358. doi: 10.1371/journal.pone.0226358 (PMC6922353; doi:10.1371/journal.pone.0226358)
Supplement: S1 Text — (DOCX) [file pone.0226358.s001.docx]

**Material and methods**

2008 samples were placed in 10 cm^3^ plastic boxes, dried and weighed. The following analyses were then performed on these samples.The core CAN REILLE (S1 Fig) was sampled each 10 cm except at minerogenic layers.

**1 Radiocarbon dating**

Radiocarbon dating was performed at the Poznan Radiocarbon Laboratory (Poland) for the 2008 samples (13) and at the Laboratory of Chemistry of Tucson (Arizona, USA) for the 1998 samples (5).The techniques used were the A.M.S. (Poznan) and the conventional liquid scintillation methods (Tucson). Subsequently, results were calibrated using OxCal 4.1.5 software (Bronk Ramsey 2009, Reimer et al. 2009); ages are expressed in cal. BC.

**2 Magnetic susceptibility measurements**

Magnetic susceptibility (MS) measurements were performed on each sample at CEREGE (Aix en Provence, France) using a MFK1-FA (Agico). Low frequency magnetic susceptibility (Xlf) was measured at 976 Hz (sensitivity ~3 x 10^-8^ SI) and raw data were related to the density of the material. The magnetic signal is due to the presence of small magnetic grains (hematite, maghemite and magnetite) whose concentration depends on several processes (Devillers 2008, Fialova et al. 2006, Thompson and Oldfield 1986). Mineralogical composition of the watershed (Vella et al. 2014, Ghilardi et al. 2015, Oldfield 1983), pedogenetic processes (Fassbinder et al. 1990), enrichment of iron oxides by soil heating (Borgne 1955, Weston 2004) and agropastoral practices could modify the mineralogy and oxidation-reduction conditions and thus influence the magnetic properties of the sediments.

**3 Grain-size analyses**

Grain-size analyses were conducted in CEREGE. All the samples were then first heated at 450°C and mixed with an agent (0.3% sodium hexametaphosphate) in order to disperse the clay particles. The grain-size distribution was measured using a Beckman Coulter LS 13 320 laser granulometer with a range of 0.04 to 2000 microns, in 132 fractions. The calculation model (Beckman Coulter LS 13 320 software version 5.01) uses the Fraunhöfer and Mie Theory (Blott et al. 2004). For the calculation model, we used water as the medium (RI = 1.33 at 20°C), a refractive index in the range of that of kaolinite for the solid phase (RI = 1.56), and absorption coefficients of 0.15 for the 780-nm laser wave length and 0.2 for the polarized wavelengths (Buurman et al. 1996). Samples containing fine particles were diluted, so that we measured between 8 and 12% obscuration and between 45 and 70% PIDS (Polarization Intensity Differential Scattering) obscuration.

### **4 Loss on ignition (LOI)**

LOI methodology was based on Dean (1974), Bengtsson and Enell (1986) and Heiri et al. (2001) and performed at the CEREGE. Sediment samples of approximately 1g were taken each 10 cm throughout the profiles. After drying at 105°C to a constant weight, the samples were heated to 550°C for 7 h to estimate organic content.

**5 Pollen treatment and determination**

Pollen analyses were conducted on 44 samples of CAN REILLE (S1 Fig). The sporo-material was extracted using a standard method (Moore et al. 1991). The identification was carried out with a photonic microscope (x500 magnification) and with the help of the pollen reference collection of IMBE (Aix-en-Provence, France) and pollen photographic books (Reille 1992, 1995, Beug 2004). The pollen diagram is drawn based on pollen percentages calculated from a Pollen Sum (PS) including all vascular plants, except over-represented local taxa (Alnus, Salix, Cyperaceae, *Sparganium*-*Typha*, *Ranunculus*), all aquatic plants (*Myriophyllum*, *Nymphaea*, *Potamogeton*, *Utricularia*) and ferns (*Isoetes*, monolete spores, *Ophioglossum*, *Osmunda*, *Polypodium*, *Pteridium*, *Selaginella*, trilete spores). The pollen curve of nitrophilous plants corresponds to the sum of *Rumex*, Cichorioideae, Asteroideae and *Urtica*. The software C2 (Juggins 1991-2007) was used to draw the pollen diagram.

**References**

Bengtsson L, Enell M. Chemical analysis. In: Berglund BE, editor. Handbook of Holocene Palaeoecology and Palaeohydrology. Chichester: Wiley. 1986; pp. 423–451.

Beug HJ. Leitfaden der Pollenbestimmung für Mitteleuropa und angrenzende Gebiete. 2004. 542 p.

Borgne E. Susceptibilité magnétique anormale du sol superficiel. Annales de géophysique, t. 11, 1955 p. 399-419.

Bronk Ramsey C. Bayesian analysis of radiocarbon dates. Radiocarbon, 2009; 51(1): 337-360.

Buurman P, Pape T, Muggler RCC. Laser grain-size determination in soil genetic studies: Practical problems. Soil Science 1996; 162: 211-218.

Dean WE. Jr. Determination on carbonate and organic matter in calcareous sediments and sedimentary rocks by loss on ignition: comparison with other methods. Journal of Sedimentary Petrology, 1974; 44: 242-248.

Devillers B, 2008 - Holocene morphogenesis and land use in a semi-arid watershed, The Gialias river, Cyprus. BAR S1775 – Archaeopress, 199 p.

Fassbinder JWE, Stanjekt H, Hojatollah V. Occurrence of magnetic bacteria in soil. Nature. 1990; 343(6254): 161-163.

DOI: 10.1038/343161a0

Fialova H, Maier G, Petrovsky E, Kapicka A, Boyko T, Scholger R. Magnetic properties of soils from sites with different geological and environmental settings. Journal of Applied Geophysics. 2006; 59: 273-283.

Ghilardi M, Cordier S, Carozza JM, Psomiadis D, Guilaine J, Demory F, et al. The Holocene fluvial history of the Tremithos River (south central Cyprus) and its linkage to archaeological records. Environmental Archaeology: The Journal of Human Palaeoecology. 2015; 20(2): 184-201.

DOI: 10.1179/1749631414y.0000000057

Heiri O, Lotter AF, Lemcke G. Loss on ignition as a method for estimating organic and carbonate content in sediments: reproducibility and comparability of results. Journal of Paleolimnology. 2001; 25: 101–110.

Juggins S. C2 Software for ecological and palaeoecological data analysis and visualisation User guide Version 1.5. Newcastle: University of Newcastle. 1991-2007.

Moore PD, Webb JA, Collinson ME. Pollen Analysis, 2nd edition. Oxford: Blackwell Scientific Publications. 1991 216 pp.

Oldfield F The role of magnetic studies in palaeohydrology. In Gregory K.J. editor. Background To Palaeohydrology: A Perspective. Chichester: Wiley.1983.

Reimer PJ, Baillie MGL, Bard E, Bayliss A, Beck JW, Blackwell PG, et al. -IntCal09 and Marine09 radiocarbon age calibration curves, 0–50,000 years cal. BP. Radiocarbon. 2009; 51-4: 1111-1150.

Reille M. Pollen et spores d’Europe et d’Afrique du Nord, supplément 1. Marseille: Laboratoire de Botanique historique et de Palynologie. 1995.

Reille M. Pollen et spores d’Europe et d’Afrique du Nord. Marseille: Laboratoire de Botanique historique et de Palynologie, 1992.

Thompson R, Oldfield F. Environmental magnetism. London: Allen & Unwin. 1986.

Vella MA, Ghilardi M, Cesari J, Leandri F, Pêche-Quilichini K, Demory F, et al. Evolution morpho-sédimentaire des marais de Canniccia à l’Holocène récent: implications paléoenvironnementales pour l’occupation du site d’I Calanchi/Sapar’Alta entre le Néolithique final et l’âge du Bronze final. In: Sénépart I, Léandri F, Cauliez J, Perrin T, Thirault E, editors. Chronologie de la Préhistoire récente dans le sud de la France. Actualités de la Recherche. Actes du colloque 10e Rencontres Méridionales de Préhistoire Récente (Porticcio, 18-20 Octobre 2012). Toulouse: Archives d’Ecologie Préhistorique. 2014; pp. 351-360.

Weston DG. The influence of waterlogging and variations in pedology and ignition upon resultant susceptibilities: a series of laboratory reconstructions. Archaeological Prospection. 2004; 11(2): 107-120.

DOI: 10.1002/arp.230
