# Supplementary figures and images for: Early impact of agropastoral activities and climate on the littoral landscape of Corsica since mid-Holocene
Source: PLoS One. 2019 Dec 19;14(12):e0226358. doi: 10.1371/journal.pone.0226358 (PMC6922353; doi:10.1371/journal.pone.0226358)

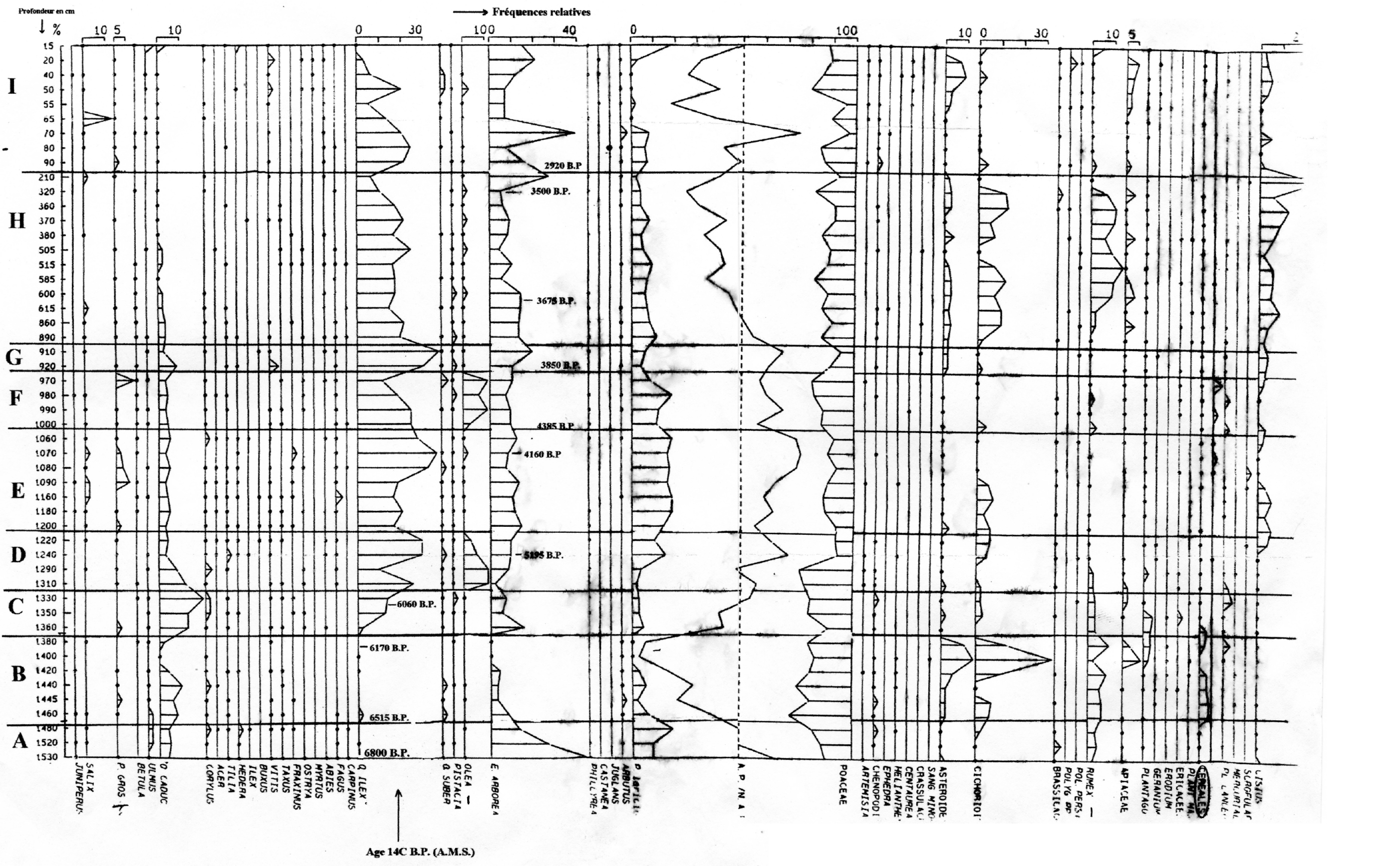

Supplement: S1 Fig — Reille 1998. (TIF) [file pone.0226358.s002.tif]
