# Supplementary material for: Early impact of agropastoral activities and climate on the littoral landscape of Corsica since mid-Holocene
Source: PLoS One. 2019 Dec 19;14(12):e0226358. doi: 10.1371/journal.pone.0226358 (PMC6922353; doi:10.1371/journal.pone.0226358)
Supplement: Attachment — Submitted filename: Vella et al _Plos One Response to Reviewers.docx [file pone.0226358.s003.docx]

Dear academic Editor, and dear anonymous reviewers,

I would like to thank you personally for your attentive reading of the article that my research team and I have proposed for publication in Plos One. I also thank you for your interest in this innovative multidisciplinary approach on past societies/environments interactions. We have taken into account all your observations, corrections and addition of bibliographical references. The general and more specific remarks you have given us allow us to significantly improve the quality of our article. We also hope that our work will make a significant contribution to the knowledge of the Mediterranean environments.

Before responding point by point to each of the reviewers, we would like to respond more generally to your comments. As you have rightly pointed out, we have sought to clarify certain points in our manuscript.

The chrono-cultural context of our study has been clarified. First in the title highlighting that the mid Holocene constitutes the upper temporal limit. Then in the text, changing the title of some parts but also adding the chronological boundaries of events identified by palynology and geomorphology.

The phases of detritism have been rectified to better correspond to the specific environment of the Canniccia pond (D1 to D4 and not D5). In the same way the presentation of the different environments (D and T) has been homogenized in order to simplify the understanding.

The missing bibliographical references that you pointed out to us were of great help for our study and allowed us to refine our chronology of the domestication phases of the olive and the vine. They also allowed us to better understand island cultural processes but also to include Corsica in a more regional, Mediterranean-wide context.

Finally, we made a last proofread to improve the English expression

Here is, in the following pages, the answers to the corrections made in the manuscript.

Thank you very much for your participation to our work, I hope to meet you at an international event around the Mediterranean Worlds.

Best regards

Marc-Antoine Vella

**Academic Editor**

Andrea Zerboni, Ph.D

***1.*** *Please ensure that your manuscript meets PLOS ONE's style requirements, including those for file naming.*

We changed our organization using the heading, reference and citations using the PLOS ONE style requirements.

We named our files: 'Vella et al_PlosOne' then

'Response to Reviewers'

'Revised Manuscript with Track Changes'

'Manuscript'

'Revised Figures with Track Changes'

'Figures'

'S1 Text. Material and Methods with Track Changes'

'S1 Text. Material and Methods'

'S2 Permission to publish BRGM'

'S3 Permission to publish IGN'

For unchanged elements of our paper

'S1 Figure. Reille Unpublished 1998'

'Highlights'

We added also the figure as a separate files named Vella et al_Plos One Fig 1 to 11.

***2.*** *We note that Figures 1, 2 and 11 in your submission contain copyrighted images.*

We specified, in the figure 1, 2 and 11 caption text, that the figure is similar but not identical to the original image and is therefore for illustrative purposes only. We referenced the origin of the data in the same way as observed in:

Andrew Gillreath-Brown, Lisa Nagaoka, Steve Wolverton. 2019. A geospatial method for estimating soil moisture variability in prehistoric agricultural landscapes, PLOS ONE.

https://doi.org/10.1371/journal.pone.0220457

Where in Fig. 1 we have:

“The data for the DEM was acquired from the USGS National Map Viewer (public domain).”

First we added as supplementary information the permission to publish 'S2 Permission to publish BRGM' and 'S3 Permission to publish IGN' and we changed the captions of Fig.1, 2 and 11 in order to clarify the origin of the data illustrated in our article:

**Fig 1. Location map of the study area.** The data for the DEM (SRTM 3) was acquired from the USGS Earth Explorer Map Viewer (public domain). The data for the geology was acquired from the BRGM (Bureau de Recherches Géologiques et Minières) Map Viewer (public domain), modified. **A: Corsica within the western Mediterranean area.** **B: Topographic map of Corsica.** Reference site: b. Bastani, c. Creno, dS, Étang del Sale, f. Fango, s. Saleccia, Sp, Spizicciu. **C: Location and geology of the Taravo drainage basin.**

**Fig 2. Sites implantation within the Lower Taravo Valley.** The data for the DEM (BdAlti®) and orthorectified aerial pictures was acquired from the IGN (Institut National de l’Information Géographique et Forestière; ®IGN2019) Map Viewer (public domain). Elevation contours (10 m) are derived from ®BdAlti25 data. The data for the geology was acquired from the BRGM Map Viewer (public domain), modified. **A: Simplified geology of the Lower Taravo Valley and location of Fig. 2B. B: Orthorectified aerial picture of Canniccia marshes.**

**Fig 11. Palaeogeographic reconstruction of the Canniccia marshes evolution for the last 5000 years. A: ~3000 - 2200 BC (Late Chalcolithic); B: 2300 - 1200 BC (Late Chalcolithic to Middle Bronze Age); C: 200 - 1000 BC (Late to Final Bronze Age); D: 1000 BC – 500 AD (Final Bronze Age to Roman times).** The data for the DEM (®BdAlti25 data) was acquired from the IGN Map Viewer (public domain; ®IGN2019).

***3.*** *Please include your tables as part of your main manuscript and remove the individual files.*

The tables have been added to the main manuscript.

***4.*** *Please amend your list of authors on the manuscript to ensure that each author is linked to an affiliation.*

We checked the authors list and their affiliation and changed some elements in order to meet PLOS ONE requirements.

***5.*** *Please include a separate caption for each figure in your manuscript.*

A separate caption of each figure has been included in the manuscript.

***6.*** *Please ensure that you refer to Figure 8 in your text as, if accepted, production will need this reference to link the reader to the figure.*

We added the reference to the Figure in the text.

***7.*** *Please include captions for your Supporting Information files at the end of your manuscript.*

A caption of each Supporting Information files has been included at the end of the manuscript.

**Reviewer #1**

**Main comments**

***1)*** *As the paper focuses on the early impact of human activities and climate on the landscape during the mid-Holocene, it would be useful to briefly describe the environmental setting/plant landscape shortly before the stable human occupation of the area during the Chalcolithic Age. For this purpose, the Authors could add/compare in the discussion the unpublished data of other (few) samples from CAN REILLE prior to 3000 BC (S1 Figure). The Authors may wish to consider this comparison in order to investigate the vegetation changes at the passage from wild to human environments (see for example: Mercuri et al. (2013) Quaternary International 303: 24-42).*

OK. Done at the beginning of chap. 5.3.

***2)*** *Regarding the methodological section, I suggest the Authors specify the main plant taxa indicators of agropastoral activities; except for the well-known crop species, pasture plants are not so commonly known (in particular, for readers not familiar with botany, clarify the ‘pastoral’ meaning of the nitrophilous plants listed in the caption of Fig. 7).*

See in SI Text: Material and Methods. In chap. 5, material & method, we have add this sentence: “The pollen curve of nitrophilous plants corresponds to the sum of *Rumex*, Cichorioideae, Asteroideae and *Urtica*”.

***3)*** *Please, reconsider the timing of the most ancient testimonies of vine cultivation in Italy (L. 455-456): the results of the research by Ucchesu et al. (2015; Vegetation History and Archaeobotany 24: 587-600) provide evidence that cultivated grapevines were present in Sardinia during the Late Bronze Age, also suggesting that the selection and domestication processes of the wild grape may have started during the Middle BA.*

“Ucchesu et al. (2015; Vegetation History and Archaeobotany 24: 587-600” added to references [148] and the dating corrected

“More recent studies attest to a primitive cultivated *Vitis vinifera* in Sardinia during the Late Bronze Age [148].”

**Minor comments:**

***1)*** *Chapter 2.2: please provide the author(s) for each plant name at the species level.*

Done.

***2)*** *Chapter 2.5: the title should be modified as in this section (and throughout the whole text) there is any specific reference to the Mesolithic Age.*

Title changed.

***3)*** *Chapter 5.1: the sub-heading titles ‘Four detritic phases…’ (L. 328), but below are presented five detritic events (L. 330); in addition, the list of these phases lacks the point 4 (I guess ‘4)’ should be before ‘D4’ at L. 339), and the point 5 refers to the opposite bank of the Taravo. The different steps of detritism should be clarified.*

Yes, it’s four detritic phases

As the Reviewer#2 suggested to describe the detritism event (D) event the same way than the phases of morphosedimentary stability (T), we removed the numbers 1) to 5). As this phase is related to storm event, I deleted this point as a fifth phase of detritism.

***4)*** *L. 407-408: The sentence is unclear: with ‘15 fires’ do you mean 15 big fire events? I could be wrong, but in the reference you quoted I didn’t see any information about ‘number of fires’; Carcaillet et al. deals with the role of fire in the past vegetation changes and confirm an increase of fire frequency since 2500 BP probably driven by the slash-and-burn agriculture. In addition, the period 2500-2000 BC is not 1000 years. Please rephrase.*

This part has been rephrased.

***5a)*** *Please, check for the accuracy of some words (e.g., L. 147-149: ‘maquis’, ‘pine forests’, ‘subsp.’ not in italics;*

“Maquis” and “Pine forest” are accepted in the scientific literature. Cf palaeoecological or ecological articles in Google Scholar.

***5b)*** *L. 246-252: the abbreviations of the authors of plant names are not in italics, and ‘Sylvestris’ not capitalized;*

Done.

***5c)*** *L. 436-437: ‘dicoccum’ instead of ‘dicoccun’, ‘Vicia ssp.’ instead of ‘Vivia ssp.’, and ‘fabaceae’ with the first letter capitalized);*

Done.

***5d)*** *numbers/dates (e.g., L. 212: add the dash between 1400 and 700, and change ‘BC’ to ‘AD’; L. 631: ‘1350’ instead of ‘135000’);*

Genoese Period: 1300-1400 AD, corrected.

L 631 Yes, modified.

***5e)*** *graphic symbols (e.g., L. 332: comma instead of bracket after 3000 BC; L. 367 and L. 369: change ‘>’ to ‘<’ and vice-versa).*

L 332. We removed 2200 BC so I kept the bracket.

L 367 and l 369. Yes, changed.

***6)*** *Table 2: the abbreviations of the authors of plant names are not in italics; dicoccum’ instead of ‘dicoccun’, and ‘Sylvestris’ not capitalized.*

Done.

***7)*** *Table 5: in LPAZ 10, ‘Olea’ should be in italics.*

Done.

***8)*** *Fig.1 caption: please specify that b. Bastani, c. Creno, etc… are reference sites.*

We precised this in the figure caption.

**Reviewer #2:**

**General considerations**

**1)** *Firstly, the title is unclear: it introduces “Early impact” and “Mid-Holocene period”, while the paper considers a period from 5000 BP (3000 BC) to present day, namely from the transition between Mid-Holocene and Late Holocene and during the whole Late Holocene. Then, much would be gained with a more clearly exposed aim of the paper.*

Yes, our paper focus much more on a period from 3000 BC. So, we choose to change the title from “…during Mid-Holocene.” to “…since Mid-Holocene.”.

**2)** *p. 8, l.349, the authors wrote “A detrital event D1 (around 3000 BC) is not recorded as major climatic events in the Western Mediterranean”. Wouldn’t it be more accurate to write “The detrital event D1 (around 3000 BC) in the Canniccia marshes does not correspond to any major climatic events recorded in the Western Mediterranean*

Ok, changed

“The detrital event D1 (around 3000 BC) in the Canniccia marshes does not correspond to any major climatic events recorded in the Western Mediterranean.”

**3)** *Period names are not standardized. The term “Late Holocene” seems more commonly used than the “recent Holocene Epoch” the authors choose at the beginning of the text. Thereafter, “Upper Holocene” is found as well.*

Period name « recent Holocene Epoch » and « upper Holocene » changed for « late Holocene ».

**4)** *In the first discussion paragraph (5.1), it would be clearer to describe the Detritism event (D) in the same way than the Phases of Morphosedimentary stability (T).*

In order to describe the detritism event (D) event the same way than the phases of morphosedimentary stability (T), I removed the numbers 1) to 5).

**Specific comments (by page/line numbers):**

**1)** *p.2 l. 56 to 58: The references cited by the authors did not attest to the presence of RCC (identified and published by Mayewski et al. 2004 at the hemispheric scale), but they highlight or document the regional/local effect of these RCC.*

Mayewski et al. 2004 added in references [20].

**2)** *l. 72-73: remove “recent Holocene” if the considered period is …” since the Last Glacial Maximum”*

“recent Holocene” removed.

**3)** *l. 84: Please explain why “archaeological research programs”… “required more paleoenvironemental analysis”.*

“as few cross-combined approaches were realized on main archaeological sites” added

**4)** *l. 86: “(“ missed*

“(“ added.

**5)** *p.3 l. 147 to 148: “maquis” and “pine forest” might be regular not italic.*

Checked.

**6)** *p.4 l. 135: “… along with maquis such as…”, add species after maquis.*

Done.

**7)** *p. 4/5. The paragraph 2.4 would be more relevant and useful for the reader with a critical review of the available data: date of the analysis, location, chrono-stratigraphical control of the sequences… (ref. [55] Reille et al. 1997, is about Late Glacial). A chronological phases description would be more appropriate than the somewhat “oldfashion” chronozones.*

For this paragraph, we have chosen to be concise because the purpose of our article is not to make a review. For this reason, we do not think it is appropriate to make the changes requested by the reviewer. This would considerably lengthen the text.

Considering “fashion”, we are not sure “fashion” exist in science ...

**8)** *p.5 l. 212: remove “1300-1400”*

Genoese Period: 1300-1400 AD, corrected.

**9)** *p.6 l. 292: Please precise which application of Blaauw you used (Clam, I think so?) and which version. Idem on Fig. 3*

“CLAM” R package (Blaauw, 2010) of R 2.13.2 software, See Also in Fig 3.

**10)** *l. 292-295: This sentence is unclear for me. What are the dates between brackets, the earliest radiocarbon dating for each sequences?*

Sentence precised.

**11)** *p.7 and Tab. 5: it would be nice to provide here the chrono-stratigraphic boundaries of the LPZ, and to precise the way you called the LPAZ. It is strange to begin a sequence with a subzone 5c.* On table 5: remove “7:” and “10:”

The numbering of the LPAZ begins at 5 because the LPAZs 1 to 4, which correspond to the bottom of the pollen record, are not published in this article.

Chronological boundaries added to table 5 (new colum)

**12)** *p.7 l.323: You talk about “four cores” that “highlight four phases of intense detritism”. Didn’t you studied the sediments of only CAN 1 to 3?*

Yes, changed “four” to “three”

**13)** *l.326: Isn’t it Fig. 8 instead of Fig. 9?*

Yes

**14)** *l.332: What is: “. 2200 BC)”?*

2200 BC)” deleted

**15)** *l.339: “4)” is missing*

As you suggested to describe the detritism event (D) event the same way than the phases of morphosedimentary stability (T), we removed the numbers 1) to 5).

**16)** *p. 8 l.351-352: more accurately, “An event that was coeval with D2 is also observed…..”*

Ok, changed

**17)** *l.378: What is D5?...*

Corrected

**18)** *p. 9 l.407-408: please be more precise, the fire history derives from the Creno sequence study.*

This part has been rephrased

**19)** *l.409: “It attests to the intensity of Chalcolithic and Early Bronze Age agropastoral activities on vegetation”. This rather might be an argued assumption.*

This part has been rephrased

**20)** *l.422/432: the references about Olea europaea in the Mediterranean are too old, and then the dating is not up to date. Please see for example: Carrion et al. QSR 29, 2010; Breton et al. C.R. Biologie332, 2009 or Newton et al. VHA, 2013.*

Dating corrected and the following references have been added

133. Carrion et al. QSR 29, 2010

134 Breton et al. C.R. Biologies 332, 2009

135. Newton et al. VHA, 2013

“More recent studies demonstrated that during the Neolithic in the western Mediterranean, *Olea* sp. became dominant in the coastal plant formations [133]. They also revealed several domestication centres [134] and underlined that during the Late Bronze Age, the role of wild olive populations in the plant economy was important [135]”

**21)** *l.438: A diversity of agricultural…*

changed

**22)** p.10 l.472: the decrease of arboreal pollen (instead of the frequencies)

Yes, modified

***23)*** *l.488: beginning the sentence with “During Roman Times…” is not consistent with what you reminded just above about “the age model of the last 2000 years is weak…. the correlations that we propose with the cultural phases are hypothetical…”*

We have nuance our words by adding “Probably…” at the beginning of the sentence.

**23)** *p.12 l.567-572: the authors assume that, in the Lower Taravo Valley, “important landscape changes … may have precipitated significant sociocultural transformations”. What about the opposite? Isn’t it possible to also discuss the hypothesis that “significant sociocultural transformations have precipitate important landscape changes”. What is in good accordance with the last sentence of the paragraph “Agropastoral activities may have contributed to soil erosion…”*

Around 1.2 ky BC, the entire West Mediterranean undergoes a CCR period. The societal transformations (Agriculture to pastoralism), already under way since the beginning of the Bronze Age, are thus accelerated and lead to an acceleration of the transformation of the landscape.

We changed the sentence to reflect the interconnection of these two transformations.

**24)** *p. 13 l.601: Cerealia instead of cerealia*

changed

**References**

**1)** *p. 22 l. 1047: 1961 (6 is missing)*

6 added

**Tables and Figures:**

**1)** *Tab.3 The bibliographical reference is wrong: [120] instead of [122].*

In Tab. 2[121] with the new references we added

**2)** *Fig. 7 The “Nitrophilous plants” curve actually sums all the apophytes: nitrophilous and ruderals. I do not understand why some taxa that are in this “Nitrophilous plants” curve, such as Asteroideae sum of Rumex, are displayed alone as well. It makes the diagram unclear.*

We precised your first observation in the figure caption

We display *Rumex* and Asteroideae in the pollen diag to indicate readers the importance of those taxa in the “nitrophilous plants” curve.

**3)** *Fig. 10 Nice synthetic figure. Just two remarks: 1) samples without pollen have got values, despite the white background, 2) the first “Occupation phases” graph, just after “Nitrophilous plants” curve, does not have an header (Corsica ?).*

1) The values that appeared in the figure have been deleted and do not appear any more.

2) We synthetized the two occupation phase graph in order to make the figure clearer.

**4)** *Fig. 11 A and B: text in black on dark grey is unreadable*

We changed the color to white in order to make the text more readable.
